# The African Health Profession Regulatory Collaborative for Nurses and Midwives

**DOI:** 10.1186/1478-4491-10-26

**Published:** 2012-08-29

**Authors:** Carey F McCarthy, Patricia L Riley

**Affiliations:** 1 Division of Global HIV/AIDS Center for Global Health, Centers for Disease Control and Prevention, 1600 Clifton Road, , MS-E41 Atlanta, GA, 30333, USA; 2 Senior Technical Advisor Division of Global HIV/AIDS Center for Global Health Centers for Disease Control and Prevention, 1600 Clifton Road, MS-E41 Atlanta, GA, 30333, USA

**Keywords:** Health workforce, Regulation, Health profession, Human resources for health, Collaborative, Global health, Regional approach, Strengthening, Nursing, Midwifery

## Abstract

**Background:**

More than thirty-five sub-Saharan African countries have severe health workforce shortages. Many also struggle with a mismatch between the knowledge and competencies of health professionals and the needs of the populations they serve. Addressing these workforce challenges requires collaboration among health and education stakeholders and reform of health worker regulations. Health professional regulatory bodies, such as nursing and midwifery councils, have the mandate to reform regulations yet often do not have the resources or expertise to do so. In 2011, the United States of America Centers for Disease Control and Prevention began a four-year initiative to increase the collaboration among national stakeholders and help strengthen the capacity of health professional regulatory bodies to reform national regulatory frameworks. The initiative is called the African Health Regulatory Collaborative for Nurses and Midwives. This article describes the African Health Regulatory Collaborative for Nurses and Midwives and discusses its importance in implementing and sustaining national, regional, and global workforce initiatives.

**Discussion:**

The African Health Profession Regulatory Collaborative for Nurses and Midwives convenes leaders responsible for regulation from 14 countries in East, Central and Southern Africa. It provides a high profile, south-to-south collaboration to assist countries in implementing joint approaches to problems affecting the health workforce. Implemented in partnership with Emory University, the Commonwealth Secretariat, and the East, Central and Southern African College of Nursing, this initiative also supports four to five countries per year in implementing locally-designed regulation improvement projects. Over time, the African Health Regulatory Collaborative for Nurses and Midwives will help to increase the regulatory capacity of health professional organizations and ultimately improve regulation and professional standards in this region of Africa. The African Health Regulatory Collaborative for Nurses and Midwives will measure the progress of country projects and conduct an annual evaluation of the initiative’s regional impact, thereby contributing to the global evidence base of health workforce interventions.

**Conclusion:**

The African Health Regulatory Collaborative for Nurses and Midwives is designed to address priority needs in health workforce development and improve regulation of the health workforce. This model may assist others countries and regions facing similar workforce challenges.

## Background

Beginning with the World Health Report 2000, landmark publications have firmly established the importance of the health workforce in advancing population health [1-6]. The shortage of human resources for health (HRH) in sub-Saharan African countries severely limits the ability of countries to meet national health targets and global health goals [7-9]. Compounding the insufficient number of health workers (defined by the World Health Organization as less than 2.5 health workers per 1000 persons), is the lack of appropriate skills, competencies, and education necessary for this workforce to deliver adequate care to the populations they serve [3,10-12]. In response to these shortcomings, global health policy makers identified regulatory actions, such as professional licensure, continuing professional development (CPD) of health workers, and accreditation of educational institutions as key interventions for strengthening the health workforce in low resource countries [3,10,11,13-15]. Implementing these key regulatory interventions requires collaboration among national stakeholders, including the health worker regulatory bodies, professional associations, and health educators [10,11,13].

Guidelines on task-shifting and suggestions for regulatory reform have been provided by the donor community and external policy-making bodies. The collaboration needed to implement this reform is often inadequate [10,11,16]. Furthermore, local health worker regulatory bodies most impacted by these recommendations, such as nursing and medical councils, often lack the capacity, resources, or expertise to enact the suggested reforms [16,17].

**Figure 1:**
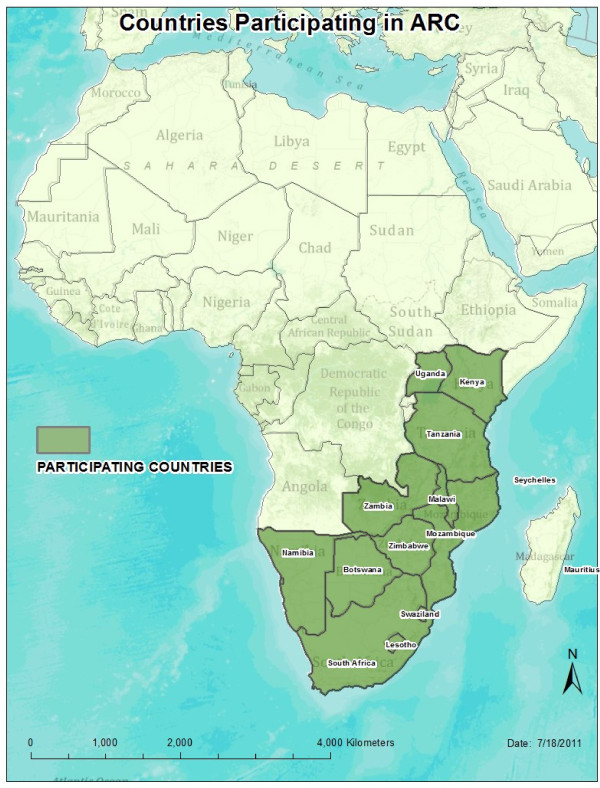
Map of countries participating in the African Health Profession Regulatory Collaborative for Nurses and Midwives (ARC).

In 2011 the United States Centers for Disease Control and Prevention (CDC) began an innovative four-year initiative to increase the collaboration among national stakeholders and help strengthen the capacity of health professional regulatory bodies to reform national regulatory frameworks. The African Health Profession Regulatory Collaborative for Nurses and Midwives (ARC) convenes leaders responsible for regulation from 14 countries in East, Central and Southern Africa (Figure 1) for a high profile, local country collaboration to assist countries in implementing joint problem-solving approaches that target national issues affecting the health workforce. The following discussion describes ARC and discusses its importance in implementing and sustaining national, regional, and global workforce initiatives.

### The African Health Profession Regulatory Collaborative for Nurses and Midwives (ARC)

ARC is a partnership between the CDC, Emory University, The Commonwealth Secretariat, and the East, Central and Southern African Health Community through the East, Central and Southern African College of Nursing (ECSACON) designed to increase the regulatory capacity of health professional organizations and ultimately improve regulation and professional standards in the east, central and southern Africa (ECSA) region. The objectives of this four-year initiative are to 1) ensure nursing and midwifery practice standards are harmonized in the ECSA Region and aligned with global standards, 2) ensure national nursing and midwifery regulatory frameworks are updated and reflect nationally approved practice and educational reforms, 3) strengthen the capacity of the health professional councils within the ECSA Region to conduct key regulatory functions and, 4) establish a sustained consortia of African nursing and midwifery leaders in practice and regulation.

The framework of ARC is adapted from the Institute for Healthcare Improvement’s (IHI) Breakthrough Series© ‘clinical collaborative’ model [18]. Under the IHI model, clinical teams from healthcare institutions are convened, along with topical experts, to address a pressing quality improvement issue. Teams plan their improvement approach, design measurement strategies, and meet regularly to report on progress, discuss challenges, and receive feedback from peers and experts. In a similar fashion, ARC convenes regional and international regulation experts and national nursing and midwifery regulation leaders from ECSA countries to address important regulatory issues impacting professional practice.

In February 2011, ARC launched this regional initiative in Nairobi, Kenya, during an invitational meeting of the 14 national regulatory teams. These leadership teams consisted of the chief nursing officer, the registrar of the nursing and/or midwifery council, the president of the professional nursing and/or midwifery association, and a representative of nursing and/or midwifery academia. Each country team developed a plan to address a locally identified regulation issue. Issues identified included developing a CPD system, revising the nursing scope of practice, or revising midwifery education standards. Ten country teams submitted project proposals for national regulation improvement projects; ARC selected five proposals for funding (up to US$10 000 each) through the end of 2011. The selected proposal topics for 2011 included CPD (Lesotho, Malawi, Swaziland), and revising nursing and midwifery legislation (Mauritius and Seychelles). Throughout 2011, ARC convened the selected country teams twice to present progress on their regulation improvement projects and share challenges and lessons learned with their peers and experts. Each ARC-funded country team is responsible for accomplishing an established set of deliverables, representing measurable improvements to national nursing and midwifery regulation. In June 2012 a new ARC annual cycle will begin with a meeting of fourteen country leadership teams and the opportunity to again develop and submit national regulation improvement projects.

## Discussion

In contrast to top-down guidelines and policy recommendations, the ARC approach to addressing health worker regulation issues begins with supporting country-driven priorities and fostering collaboration among regulation stakeholders. ARC’s framework, based on a proven model for advancements through collaboration, provides a regional forum for countries to share approaches with each other and learn from similar experiences, challenges and successes. It is hoped that south-to-south collaboration of ARC will increase each year as countries funded in earlier cycles share lessons learned with countries experiencing similar workforce challenges. This peer-to-peer learning is designed to create long-term capacity among the regulatory leadership of the ECSA nations. The collaboration among the national regulation stakeholders will also help ensure that improvements to regulation are integrated into key institutions in the health and education sectors which are critical to sustaining health workforce improvements [10,11]. The regional focus of ARC should allow for a rapid scale-up of successful regulatory reform. ARC’s partnership with ECSACON provides a regional platform from which to coordinate and harmonize nursing and midwifery regulation.

ARC not only advances regional priorities for nursing and midwifery regulation, but also supports global priorities as articulated by the World Health Organization’s 2011–2015 Strategic Directions for nursing and midwifery, such as collaboration among government, professional, and education stakeholders and reforming nursing and midwifery education and practice regulations [19,20]. Further alignment with global standards and guidelines is ensured through attendance at and participation in ARC meetings by representatives from the International Council of Nurses, the International Confederation of Midwives, the World Health Organization’s Regional Office for Africa, and others. Furthermore, ARC will measure the progress and impact of country projects as well as conduct an annual evaluation of the ARC approach, thereby contributing to the global evidence base of HRH interventions.

## Conclusion

Major donor and policy makers have clearly articulated global health workforce priorities requiring collaboration among stakeholders and institutional capacity within health professional regulatory bodies. ARC institutes a country-driven, south-to-south collaborative approach that empowers local and regional regulatory leadership to address issues affecting the largest component of the health workforce in this region. The ARC model for strengthening the nursing and midwifery workforce in East, Central and Southern Africa is adaptable to other health care cadres and other regions struggling with similar health workforce challenges.

## Abbreviations

ARC, The African Health Profession Regulatory Collaborative for Nurses and Midwives; CDC, Centers for Disease Control and Prevention; CPD, continuing professional development; ECSA, East Central and Southern African; ECSACON, East Central and Southern African College of Nursing; HRH, human resources for health; IHI, Institute for Healthcare Improvement.

## Authors’ contributions

CM drafted and revised the manuscript. PR provided critical revisions to the manuscript for intellectual content. All authors read and approved the final manuscript.The findings and conclusions in this report are those of the authors and do not necessarily represent the official position of the Centers for Disease Control and Prevention.

## Competing interests

The authors declare they have no competing interests.
